# The impact of teaching approach on horse and rider biomechanics during riding lessons

**DOI:** 10.1016/j.heliyon.2025.e41947

**Published:** 2025-01-14

**Authors:** Anna Byström, Agneta Egenvall, Marie Eisersiö, Maria Terese Engell, Sigrid Lykken, Susanne Lundesjö Kvart

**Affiliations:** a Department of Anatomy, Physiology and Biochemistry, Faculty of Veterinary Medicine and Animal Science, Swedish University of Agricultural Sciences, Uppsala, Sweden; b Department of Clinical Sciences, Faculty of Veterinary Medicine and Animal Science, Swedish University of Agricultural Sciences, Uppsala, Sweden; c Equine Teaching Hospital, Department of Companion Animal Clinical Sciences, Faculty of Veterinary Medicine, Norwegian University of Life Sciences, Oslo, Norway; d Division of Equine Studies, Department of Anatomy, Physiology and Biochemistry, Faculty of Veterinary Medicine and Animal Science, Swedish University of Agricultural Sciences, Uppsala, Sweden

**Keywords:** Rider education, Equestrian feel, Riding teacher, Equestrian coach, Rein tension, Kinematics, Practical knowledge

## Abstract

Riding relies on embodied and practical knowledge and is predominantly taught during practical lessons. Effective teaching is dependent on relevant instructions and evaluation from the riding teacher or trainer. The aim was to investigate how riding instructions affect horse and rider motion and rein tension during transitions between walk and trot.

Two Swedish (S1, S2) and two Norwegian (N1, N2) riding teachers, and five riders per location participated. Each rider rode two horses, 40 lessons total. Videos, horse and rider kinematics and rein tension were recorded. The teachers were interviewed, teacher-student interactions were analysed using conversation analysis. Biomechanical data were analysed in mixed models.

S1 and N2 spent about a third of their lessons preparing the students while S2 and N1 began with straight-line walk-trot transitions early on. With S1 and N2, maximum rein tension before and during down-transitions was lower than with S2 or N1. S2 and N2 focused relatively more on the walk, asking the riders to count each walk stride or focus on the rhythm. With S2, the timing between up-down movements of the withers and croup in walk was closest to the ideal 25 % (16–17 % vs. 8–14 % for the others, p < 0.05). With N2, horses showed the best walk hind limb protraction consistency (stride-to-stride difference 1.2–1.3° vs. 1.5–1.7°, p < 0.05).

The results show that experienced riding teachers can have a consistent influence on a group of students and indicate that lesson design impacts rein tension. Experiences from this study can be used to inform teaching of riding, for the benefit of both riders and horse welfare.

## Highlights

• Unique interdisciplinary study of lessons in horseback riding.
• Combining quantitative measurements, interviews and video recordings provided new insights about riding lessons.
• Riding teachers can influence the movement pattern of the riders and their horses.
• More preparation of the students before the transitions led to lighter rein aids.

## 1 Introduction

Riding is an activity that involves embodied and practical knowledge [1]. As such, specific conditions are required to facilitate learning. In addition, during riding lessons there are three individuals present, teacher, rider and horse. The teacher normally gives instructions to the rider on both what exercises to do and how to make the horse perform them [1]. When riding horses it is common to use negative reinforcement, i.e. pressure signals that are released when the horse behaves as intended [2]. When using negative reinforcement optimal timing is key, both for performance and for horse welfare. The rider's ability to act timely is an integral part of equestrian feel, which is a central concept in rider education [3,4]. Effective teaching of equestrian feel is dependent on timely instructions and relevant evaluations from the riding teacher or trainer [1]. This suggests that riding instructions could have a direct influence on horse and rider performance, and even horse welfare, which makes this an important topic to study. For single instructions, this influence is likely largest for tasks that are more difficult or require extra precision, for example, transitions. However, it is also possible that overall factors, such as lesson structure, have a bigger impact than how specific instructions are expressed.

Interactions between participants in an activity can be explored for example using conversation analysis (CA). The advantage with the method is that all actions (verbal as well as embodied) are taken into account in the analysis, which is particularly interesting when studying teaching of embodied activities [[5], [6], [7]]. From a conversational analytic perspective [8], different categories of instructions have been identified. One type of instructions is used in teaching how to perform specific activities, for example, how to make a transition between walk and trot. A second type entails directives or orders on what to do in an (ongoing) educational situation or elsewhere, for example ‘go to the left’. A third category is how instructions are used in written manuals, for example, in the equestrian literature. Further, all types of instructions can be designed as corrections or evaluation of the instructed action. Instructions can also be repeated and developed until students display understanding [8]. In CA, each action performed by a participant, including both embodied actions and verbal communication, is made relevant in relation to previous actions [9], paying close attention to the sequential and temporal organisation of the participants' actions [9]. In the context of a riding lesson, this can describe how the participants on a turn-by-turn basis co-create instructional sequences that usually include an instruction followed by instructed action(s), and finally an evaluation of the instructed action. This is also defined as an Initiation-Response-Evaluation (I-R-E) sequence [10]. Since riding is a mobile activity, both the current activity and the distance between the participants influence when such instructional sequences occur [11]. There are typically moments, limited in time and space, when the riding teacher and an individual student co-create what Lundesjö Kvart [11] calls instructional spaces, within which instructional I-R-E sequences can take place. Instructional spaces naturally form when the rider is instructed on precise and time-sensitive tasks, such as transitions, especially if performed near the teacher. For example, during an instruction to the group to prepare for trot, one rider (having too long reins) approaches the teacher, the teacher interrupts herself and tells the individual rider to shorten her reins which the rider immediately does and get an evaluation, “good”. Then the teacher continues to instruct the group. However, previous studies on the interactions between rider and riding teacher have relied on visual observation of the participants' actions, despite that there is a number of studies on both human and equine biomechanics during horse-rider interactions, e.g. describing the horse's footfall sequence [12,13] and rein tension during transitions [14].

Horses typically walk at slow speeds, trot at intermediate speeds, and canter or gallop at faster speeds [15]. Dressage horses are, however, taught to perform their natural gaits through a wide speed range, and to perform transitions on the rider's signal rather than from natural triggers [15]. Each of the horse's basic gaits has a unique footfall sequence [16]. When performing a transition, riders usually aim for the horse to change directly from the footfall sequence of one gait to that of another gait, which is called a direct or type 1 transition [12,13]. If there are intermediate strides with a footfall sequence not typical for either gait (from here on ‘undefined‘ gait), the transition is classified as indirect or type 2 [12,13]. In dressage, the latter is considered less ideal. The movement pattern of the rider similarly differs between gaits [[17], [18], [19]]. For example, the forward-backward tilt (pitch) of the rider's pelvis has a lower range of motion (ROM) in walk than in trot. When making a transition the rider needs to adapt to the changes in the horse's movements to remain in balance. Rein tension, which is influenced by both horse and rider, displays gait-specific patterns as well [20,21], and the magnitude of rein tension changes characteristically throughout a transition depending on the gaits involved [14].

During riding lessons, the teacher's role is to give instructions and feedback to influence the horse's and rider's performance in a positive way. Video recordings of riding lessons have revealed that teachers use verbal instructions but also body language and demonstrations to communicate their instructions [[22], [23], [24]]. Teaching riding can be challenging, as the teacher must take the experience level and needs of both horse and rider into account, and since horse-rider communication is quite complex. For example, the reins can be used to ask the horse for a change in speed or direction, or to influence the horse's head-neck posture, and both horse and rider need to learn and distinguish between these different types of rein signals [25]. This makes horse-rider-teacher interactions equally complex to study. One way to fully capture the many forms of interactions that are ongoing at once is to combine qualitative analysis of the communication between rider and riding teacher with quantitative methods that can record horse and rider movements.

Within sports performance research the need for interdisciplinary studies has long been recognised, and such studies are also becoming increasingly common [26]. A proposed definition for interdisciplinary research is: any study or group of studies undertaken by scholars from two or more distinct scientific disciplines, based upon a conceptual model that links or integrates theoretical frameworks from those disciplines [27]. Within horse-rider interaction research there have been studies that combine, for example, biomechanical measurements with behaviour registrations [[28], [29], [30], [31]], or subjective performance evaluation [[32], [33], [34]]. However, the integration of theoretical frameworks between different disciplines has been relatively limited thus far. The current study attempts to take this a step further, exploring horse-rider-teacher interactions during riding lessons using a mixed methods approach, which combines qualitative and quantitative analyses. For comparison, it has been shown that a more comprehensive understanding of sport performance can be obtained through an interdisciplinary approach, using football players as example [35].

The overall objective of this study was to investigate how riding instructions affect horse and rider biomechanics in association with transitions between walk and trot, using a combination of qualitative and quantitative research methods. Transitions were considered to present a suitable level of challenge and how transitions should be performed is described in terms that can be objectively measured (clean gait before and after, minimal transitional strides, light rein signals), making it a suitable exercise for this study. Our goal was to investigate whether individual riding teachers have a consistent influence on their students when it comes to horse and rider motion patterns or rein tension magnitudes, and if so, whether this is linked to individual differences in teaching style or lesson structure. The study aimed to describe interactions between riding teacher, rider and horse within instructional spaces (qualitative analysis using CA), including differences and similarities among teachers. Secondly, the study aimed to quantify horse and rider motion pattern and the magnitude of rein tension, before, during and following the transitions, and compare these measurements to the qualitative analysis, where the hypothesis was that there would be significant differences between lessons with different riding teachers. Combining the results from these qualitative and quantitative analyses, the third aim was to explore if differences in horse and rider biomechanics and rein tension magnitude could be explained from what riding instructions that were given and when (revealed with CA) and from the overall lesson design.

## 2 Material and methods

### 2.1 Participants

The study included 10 adult riders, four experienced riding teachers, and 11 Warmblood riding horses. The riders were students at the riding teacher educational programs at the equestrian centres Strömsholm in Sweden and at Starum in Norway and were all at medium level in dressage (equivalent to US Third Level, UK Medium or Advanced Medium, German M-level). These two equestrian centres are the main facility for riding teacher education in the respective country. The riding teachers worked as instructors at the respective riding teacher educational programs, they had formal riding teacher education accredited at level 2 or 3 according to the IGEQ's (International Group for Equestrian Qualification) level system [36]. This means that they were considered by their peers to be very knowledgeable, and that they, as ‘teachers of teachers’, had had previous opportunities to reflect on how and when to give instructions to achieve the best learning outcome for horse and rider. The horses were school horses at the equestrian centres and were educated to medium or advanced level in dressage. The horses were between 6 and 19 years old, median 11 years. Withers height ranged between 162 and 173 cm, median 167 cm. All horses were screened for lameness at the start of each data collection day, through visual assessment while trotting in hand on a straight line, by a veterinarian experienced in equine orthopaedics. All horses wore their own dressage saddle. Bits and nosebands used are detailed in S1 Table. All horses wore the same equipment throughout the study, except that one horse wore single-jointed rubber snaffle the first day and a double-jointed snaffle the second day.

Ethical permission was sought from Uppsala County Ethical Board for Animal Experiments for the horses' participation in the Swedish part of the data collection (permit reference number 5.2.18–8228/18), as the National Equestrian Centre at Strömsholm is registered as an animal experimental facility. Animal or human ethical permission was otherwise not needed according to Swedish (Ethical permission legislation/Etikprövningslagen §3–4) and Norwegian (Regulation for use of animals in research/Forskrift om bruk av dyr i forsøk §2; Law about medical and health hazardous research/Lov om medisinsk og helsefaglig forskning $1–4) laws and regulations, considering this was a non-invasive study with no additional risk for neither horses nor humans above their normal daily activities. Riders and riding teachers volunteered to participate after having received information about the study purpose and general design, including the measurements to be acquired. Both riders and riding teachers provided written informed consent for their participation, and the stable manager at each equestrian centre provided written informed consent for the horses’ participation. Participants were informed that the responsible researchers would store the collected data, including video and audio recordings, along with their first and last name and email address, but that no data containing personally identifiable information would be shared publicly. The participants were also informed whom to contact if they in the future would like to have their data deleted.

### 2.2 Data collection

#### 2.2.1 Design and data collection protocol

Data were collected during two days in each location, one week apart in Sweden and two days apart in Norway. A total of 40 lessons were recorded (Table 1). Each riding teacher participated on a single day and held two lessons with each rider. On each day, riders received lessons on two horses, one horse they were familiar with (had ridden regularly for some time, same horse both days), and one horse that they were not accustomed to (ridden only once or twice at most), a different horse each day. This yielded a partial crossover of horses and riders within each location (Table 1). The same five horses participated on both days in each location, except that in Sweden one horse had to be replaced the second day as it was lame when assessed before the beginning of the data collection (hence, a total of eleven horses were included). Before, in between and after the data collection days, horses, riders and teachers participated in their regular activities at the respective equestrian centre.

**Table 1:** Schematic overview of the data collection.

	Sweden				Norway			
Day	Lesson	Teacher	Rider	Horse	Lesson	Teacher	Rider	Horse
1	1	S1	1	1	21	N1	6	6
1	2	S1	1	2	22	N1	6	7
1	3	S1	2	2	23	N1	7	7
1	4	S1	2	3	24	N1	7	8
1	5	S1	3	3	25	N1	8	8
1	6	S1	3	4	26	N1	8	9
1	7	S1	4	4	27	N1	9	9
1	8	S1	4	5	28	N1	9	10
1	9	S1	5	5	29	N1	10	10
1	10	S1	5	1	30	N1	10	6
2	11	S2	5	5	31	N2	6	6
2	12	S2	3	5	32	N2	6	8
2	13	S2	3	11	33	N2	8	8
2	14	S2	5	2	34	N2	8	10
2	15	S2	2	2	35	N2	10	10
2	16	S2	2	4	36	N2	10	7
2	17	S2	4	4	37	N2	7	7
2	18	S2	4	1	38	N2	7	9
2	19	S2	1	1	39	N2	9	9
2	20	S2	1	11	40	N2	9	6

Horse 3 was lame on day 2 and replaced by horse 11.

The riding lessons took place in an indoor riding arena, within a marked area of 20 × 40 m. During the lessons, the horse and rider were alone in this space, but one other horse was present in the adjacent warm-up area.

The riding teachers were asked to give 20-min lessons with a focus on timing of the aids during transitions. They were asked to perform the transitions primarily along one long side, such that horse and rider would be in the field of view for the video cameras (detailed below). Further, it was requested that each horse and rider would perform at least two transitions between walk and trot, and two between trot and canter, in each direction. To avoid interfering in the teachers’ lesson plans and affect how the teachers conducted their lessons, the teachers were otherwise free to instruct the riders as they saw fit, including changes of direction, deciding on the order of the above-mentioned types of transitions, and suggesting additional exercises in between transitions as needed, e.g. using circles to improve bending and suppleness. The goal was that the lessons should resemble normal riding lessons as much as possible. This information was provided to the riding teachers both orally and summarised in written form several weeks in advance. The riders were informed about the purpose and general design of the study before volunteering to participate and received detailed information about lesson content and goal from each riding teacher at the beginning of the lesson.

#### 2.2.2 Riding teacher interviews

Each day started with a semi-structured interview with the riding teacher of the day. These qualitative interviews [37] focused on the teachers’ understanding and perceptions of how to teach riding in general, and transitions in particular, and took about 30 min. An interview guide (S1 File) was used, and the teachers were encouraged to give detailed answers. At the end of each day, a follow-up interview was made focusing on how the riding teacher perceived the instructional spaces that took place in connection with the transitions. Selected video clips were shown to help the riding teacher remember each rider. All interviews were audio recorded.

#### 2.2.3 Monitoring and measuring equipment

**Fig. 1:**
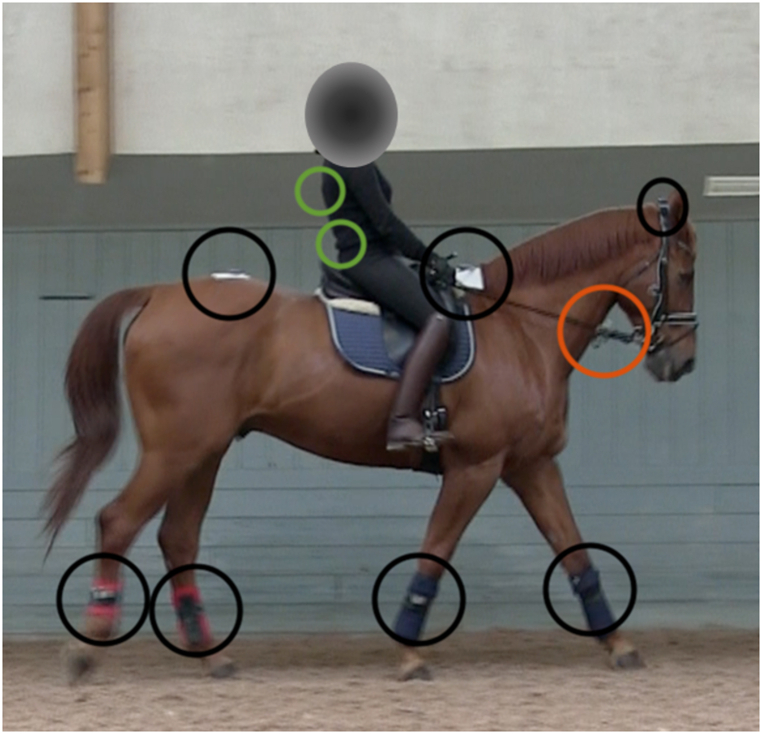
A horse and rider with measurement equipment. Black circles indicate locations of inertial measurement unit (IMU) sensors for registering the horse's movements. Green circles indicate locations of IMUs for registering the movements of the rider's pelvis and trunk (trunk data not used in the current analysis). The orange circle indicates the rein tension meters attached to the reins.

During the riding lessons, horses and riders were equipped with an EquiMoves inertial measurement unit (IMU) system (https://equimoves.nl/). It comprised 10 wireless sensors (ProMove mini), which recorded motion data, and a receiver (Inertia Gateway) connected to a laptop computer for capturing the data. The sampling rate was set to 200 Hz. The IMU sensors were automatically time-synchronised with a precision of 100 ns [38]. Sensors were placed at the horse's poll, withers (T4-T6) and croup (tubera sacrale) using adhesive tape, and laterally over the middle part of the cannons (third metacarpal/metatarsal bones) using dedicated brushing boots (Fig. 1). Sensors were additionally attached to the skin on the rider's back over the sacrum, and over the 6-7th thoracic vertebra (between the shoulder blades) using adhesive tape. The horses were also equipped with two custom-made rein tension meters, one for each rein. Each rein tension meter consisted of a load cell (range 0–500 N, weight 20 g, Futek, CA, USA) wired to amplifiers connected to an IMU that was used as A/D converter and data logger (10-bit resolution, x-io technologies, Bristol, UK). This IMU was attached to the bridle next to the EquiMoves poll sensor. The sampling rate was 100 Hz. The rein tension meters were attached to flat leather reins with leather stoppers, inserted close to the bit. This pair of reins was used during all lessons. The rein tension meters were calibrated before and after data collection on each day using ten known weights ranging from 0.2 to 10 kg.

The riding lessons were audio and video recorded using two video cameras, one capturing a side view of the opposite long side (camera 1), and one providing a frontal/rear view of the same long side (camera 2). The riding teachers wore a microphone connected via Bluetooth to camera 1 to ensure that the instructions were captured clearly. To synchronise the video recordings with the rein tension data (and thereby indirectly with the EquiMoves data) a member of the research team pulled on one rein tension meter (not affecting the horse's mouth) five times while counting out loud in front of the cameras before and after each lesson.

### 2.3 Data analysis

#### 2.3.1 Analysis strategy

By design, this study focused on transitions between gaits. The current analyses are limited to transitions between walk and trot performed on the long side in view of the cameras. The sequences of interest were identified from the video recordings as described below, and only data from those sections were included in the statistical analysis. The interviews and lesson videos, and the IMU and rein tension data, respectively, were first analysed separately by different researchers within the team, to avoid bias. Then the analysed transcripts from the interviews and the analysed interactions from videos were combined with the results for biomechanical variables, i.e. all results were reviewed as a whole.

#### 2.3.2 Analysis of interview data

The audio recordings from the interviews were first transcribed precisely to maintain authenticity. Care was taken to keep the Swedish or Norwegian wording as close as possible to each teacher's original statements. A phenomenological approach was used to analyse the transcribed material [39], where the goal is to understand how the interviewed person perceives and senses the world, not necessarily how it actually is [40,41]. The riding teachers' statements were then categorised into phenomena based on the topics they addressed. In this article the findings from the interviews were only used to aid the interpretation of the results from the lesson recordings and the biomechanical data and will not be presented in detail.

#### 2.3.3 Analysis of video recordings

All instructional spaces associated with up and down-transitions performed on the long side in view of the cameras were identified from the video-recordings. Timestamps for when the horse entered and left the long side, as well as for when transitions occurred, were recorded. The transitions were then numbered and labelled based on the gaits involved and in what direction the horse was moving around the arena. Transitions between walk and trot were selected for further analysis. Verbal and embodied interactions during the instructional spaces associated with these selected transitions were then transcribed. A typical example of such an instructional space is: the riding teacher instructs the rider to make a transition from trot to walk, the rider performs a transition and immediately after the instructed action follows an evaluation of the transition and/or instruction towards the transition back to trot, which the rider then performs (as the next instructed action) and, finally, an evaluation of the last transition or the sequence as a whole. The analysis focused on *what* aspects of the rider's and/or horse's performance that the teachers focused on in their instructions, not *how* or *when* the instructions were given, as long as it was within the analysed instructional space.

#### 2.3.4 Analysis of objective data

IMU and rein tension data were processed using custom scripts written in Matlab (version 2021b). Raw IMU data were processed to yield vertical displacement and rotation data (roll, pitch, and yaw) for each sensor [38]. Sagittal plane angular rotation, representing the forwards-backwards swinging (protraction-retraction) of the limb/cannon, was calculated for the limb IMU sensors [38]. Rein tension and EquiMoves data were synchronised using cross-correlation of the vertical translation calculated from the IMU used as rein tension data logger and from the EquiMoves poll sensor. Ground contact times for all four limbs were determined from limb sensor data using a previously validated algorithm [42]. Data were extracted for all complete strides starting and ending with ground contact of the left hind limb as well as starting and ending with ground contact of the right hind limb (i.e. there was 50 % overlap between consecutive left and right hind limb strides). Strides where the automatic stride split had failed (<5 %) were excluded. Vertical displacement data from the withers and croup sensors were subjected to frequency decomposition [43] and the phase for the frequency component with a period of two oscillations per stride (the dominating component in walk and trot, equivalent to step frequency) was determined.

For each stride, the differences between ground contact times of each hind limb and the diagonal and the ipsilateral forelimb, respectively (i.e., diagonal advanced placement – DAP, and lateral advanced placement – LAP), were calculated in percentage of stride duration. Based on DAP and LAP, each stride was classified as walk, trot, left canter or right canter, or as ‘undefined gait’ if the stride did not fulfil the criteria for any of those gaits (code in S2 File, function label_gaits). In trot, which has diagonal stance phases (e.g. left front – right hind), DAP should be near zero and LAP 50 %. In walk, a four-beat gait without suspension phase, DAP and LAP should both be 25 %, though DAP will be negative, since the footfall sequence is left fore > right hind > right fore > left hind > left fore and so on. Strides were classified as walk if DAP was between −15 and −35 % for both hind limbs, and as trot if DAP was ±10 % for both hind limbs.

Transitions between gaits were identified automatically through comparison of gait classification labels between consecutive strides (S2 File, function classify_strides). Two consecutive strides of walk or four consecutive strides of undefined gait were used as threshold to identify transitions. Up to four strides immediately before the transition were labelled as preparation, and up to four strides immediately following the transition were labelled as post-transition. Fewer than four strides were included if a transition was preceded or followed by fewer than four consecutive strides labelled with the gait the horse was transitioning from or to, respectively (e.g. labelled with walk for preparation before a walk-trot transition). Post-transition and preparation were allowed to overlap between down- and up-transitions (illustrated in S1 Fig).

For each stride, a number of discrete variables were calculated: 1) forelimb and 2) hind limb protraction-retraction ROM (range of motion), hind limb 3) maximum protraction and 4) maximum retraction; 5) consistency in hind limb protraction and 6) retraction, calculated as the absolute value of difference in hind limb maximum protraction/retraction in consecutive strides; 7) phase between withers and croup vertical displacement relative to stride duration (±0–50 %, a positive value indicates that the withers’ peaks and troughs precede those of the croup); 8) minimum, 9) median and 10) maximum rein tension averaged for left and right reins; 11) absolute difference in median rein tension between left and right reins; rider pelvic 12) roll (transversal plane rotation, i.e. side-to-side tilt) and 13) pitch (sagittal plane rotation, i.e. forwards-backwards tilt) ROM.

#### 2.3.5 Statistics

Linear mixed models (identity link) were developed in SAS (version 9.4, TS1M6), using stride-by-stride data (proc mixed SAS). Down- and up-transitions were analysed in separate models. Models were made with each of the horse and rider movement variables and rein tension variables described above as the dependent variable. Normality of the dependent variables was investigated using distribution plots and qq-plots. If deviation from normality was found, the data were transformed along the ladder of powers using Box-Cox transformation (proc transreg SAS). The fixed effects were transition phase (preparation, transition, post-transition), riding teacher (n = 4) and the 2-way interaction between phase and riding teacher. Random effects were horse, rider and transition serial number nested in lesson serial number. Additionally, number of strides per transition phase was analysed, using the same model formula except that transition serial number was not included in the random part, since each transition phase only had one data point per transition. Residuals were scrutinised to confirm normality and homoscedasticity across the range of the dependent variable. Manual backwards reduction was then used to remove non-significant terms. Least square means for phase and teacher were retrieved, and post-hoc, pairwise comparisons between teachers were made for each transition phase using the SAS option pdiff. The p-value limit for statistical significance (alpha) was set to 0.05. Correction for multiple testing was not done, since this was an exploratory study where each outcome variable was considered on its own.

## 3 Results

In the results text, the nicknames Anna (S1), Bella (S2), Cecilia (N1) and Diana (N2) are used for the riding teachers. The teachers at each location will be referred to as pair S and pair N as the study was not designed to address differences between Swedish and Norwegian riding teachers in general (only two teachers per country, no crossover of horses or riders between locations). Quotes from the teachers have been included to exemplify their actual statements; these have been translated to English but kept as close to the original wording as possible.

### 3.1 Riding teacher interviews–thoughts on teaching

The four teachers had mostly similar ideas about their role during riding lessons and general thoughts on giving instructions. Discussion and mutual understanding between teacher and rider were keywords mentioned by all teachers. “*For me, it is quite important that you should have a communication and understanding with the person that's riding, know that you are on the same page.*” (Diana). Reflecting on the lessons, all riding teachers thought it was their responsibility to prepare the riders for the exercise, and considered timely feedback (evaluation of instructed action) the most crucial for helping the riders learn to give properly timed signals to the horse. “*When you manage to time it such that I have time to give feedback in a way that the rider understands to do the same thing next time to get a good transition. So that they recognise which part of their body they used to make a good transition.*” (Cecilia).

### 3.2 Descriptive results for the transitions

During the 40 lessons, 333 transitions between walk and trot were performed on the designated long side of the arena, in view of both video cameras (93 transitions with Anna – S1, 91 with Bella – S2, 83 with Cecilia – N1, 66 with Diana – N2). In all cases, the rider entered the long side in trot and made a down-transition to walk. In most cases this was followed by an up-transition back to trot before leaving the long side. A few transitions could not be detected from the biomechanical data either because the walk sequence was very brief or due to stride split failure. This left 325 transition sequences available for further analysis, including 325 down-transitions from trot to walk (167 in left direction and 158 in right direction), and 299 up-transitions from walk to trot (153 in left direction and 146 in right direction). Following exclusion of strides with stride split failure there were no strides available for the transition phase in 9 down-transitions. For the down-transitions, this left 4518 strides with biomechanical data for statistical analysis (2439 preparation, 578 transition and 1501 post-transition). For up-transitions there were 4295 strides (1329 preparation, 610 transition and 2356 post-transition). All included strides had complete data, except that protraction-retraction data (max, min, ROM, consistency) were unavailable for one lesson.

### 3.3 Interaction between teacher and rider during the transitions

In the current context, an instructional space [11] was defined to last from when the horse and rider entered the long side until they left the long side where the transitions were performed. Within each instructional space, the interaction between the teacher and rider typically followed the I-R-E (Initiation-Response-Evaluation) sequence [10]. These interactions are characterised below for each teacher, exemplified by quotes.

Anna (S1) always asked the riders to ride on a circle around her during the first two or three transitions in each direction, and during some lessons the rider never left the circle. She asked for more energy – better engagement and more impulsion from the horse. For example, she would tell the rider to have “*a little more energy*” or to “*ride a little bit more forward*” and wanted the horse's gait to be “*short and quick*” or “*short and energetic*” in the transitions. Beside more energy, Anna addressed the riders' seat by, for example, asking the rider to “*straighten up and tighten your stomach*” and the riders' hands, especially to keep “*same tension in both reins*”. She also, to the same extent as Bella and Cecilia, wanted the horses to be straight in the transitions. Bella (S2) was the teacher that most clearly focused on counting walk strides between down- and up-transitions and wanted the riders to begin with the exercise directly from the start. She asked almost all riders to ride exactly four or five strides in walk and she often helped the riders by counting the strides out loud. Apart from counting strides, Bella had a similar focus as Anna: the horse’s energy – “*It’s not really that he should trot or walk faster, but more that you get a response*”, the rider’s seat – “*try to make it feel like you have glue under your bum so that you stay down in the saddle*”, the riders' hands - “*and slightly massaging with the bit*”, and the straightness of the horse – “*keep him straight throughout the transition*”. Cecilia (N1) also wanted the riders to begin with transitions on straight lines directly from the start. She often addressed multiple different aspects of the horse’s and rider’s performance during her lessons, with a broader focus compared to the other teachers. She gave about the same number of instructions regarding the riders' seat and hands as the other teachers, but she additionally addressed several other aspects during each transition. For example, during one lesson she targeted the following aspects across five consecutive transitions: the horse’s energy – ”*Feel there that before you ride him forward again you can make him be a little more swift*“, the rider’s gaze - ”*Look straight ahead*”, the rider’s hand – ”*Keep your hands in the same position the whole way*”, the rider’s seat – ”*Come a little forward with your upper body, look at me, you can sort of straighten out your shoulders a little forward right here at the sternum*”, and the straightness of the horse – “*Remember to give a little on the inside rein so that your horse is straight all the way through. Meet him with your outside leg so that your outside leg is like a wall to him so that he offers to go forward straight ahead*”. She was also the teacher that gave the most instructions regarding the riders' gaze as well as riders' thighs.

Diana (N2) used a long warm-up in walk, typically nearly half the lesson time, and she asked the riders to make halts from walk several times before she let her students begin with transitions between walk and trot. Diana focused less on the straightness of the horse compared to the other teachers, instead she focused more on precision in the transitions. She asked the rider to perform both down- and up-transitions at a specific point, “*we must have it at that letter*”, instead of asking for a specific number of walk strides. She also asked the riders to slow down the tempo and lower the speed – “*don't be in a hurry, keep calm*”, and addressed the horses' rhythm in walk – “*calm, calm, four-beat*”, more frequently than any of the other teachers. Overall, she was predominantly focused on the horses' actions, unlike especially Anna and Bella, who focused more on the riders.

In summary, during the 93 transitions with Anna (S1) the students received 120 different instructions, with Bella (S2) 112 instructions during 91 transitions, with Cecilia (N1) 111 instructions during 83 transitions, and with Diana (N2) 74 instructions during 66 transitions (not counting directives and short positive evaluations, e.g. ‘okay’ or ‘mm’). These numbers reflect that the teachers gave sometimes just one, sometimes multiple, instructions within an instructional space. The number of times various aspects were addressed by each teacher is summarised in Table 2.

**Table 2:** **Number of instructions per topic.** Instructions given by riding teachers during 333 instructional spaces within which transitions between walk and trot were performed (Anna 93 transitions, Bella 91, Cecilia 83, Diana 74) summarised as count per topic and teacher. Data were collected at two locations (S, N). For each teacher two lessons with each of five students were recorded (10 lessons per teacher), with partial crossover of horses and riders within location.

FOCUS	Anna (S1)	Bella (S2)	Cecilia (N1)	Diana (N2)	Total
rider's seat	21	18	19	16	74
rider's hands	18	18	19	12	67
rider's thighs			14	1	15
rider's gaze			10	1	11
straight horse	21	23	26	8	78
more energy	46	18	3	6	73
slower tempo	3	3	7	11	24
count walk strides	4	25	5	1	35
transition precision	1	6	5	10	22
horse's rhythm	6	1	3	8	18
Total all topics	120	112	111	74	417

### 3.4 Horse and rider motion pattern and rein tension variables

#### 3.4.1 General findings

The number of strides for each transition phase was similar among teachers (Table 3), except that for Cecilia (N1) the number of transition strides was slightly but significantly larger during down-transitions than for Diana (N2), and that the number of walk strides was significantly larger for Cecilia and particularly Diana, compared to Anna (S1) and Bella (S2). The latter reflects that particularly Diana often instructed the riders to remain in walk longer between down- and up-transitions compared to the other teachers. S1 Fig. shows an example of raw data and footage from one transition sequence.

**Table 3:** **Number of strides per riding teacher and transition phase**. Least square means (Est) with standard error (SE) by riding teacher (S1, S2, N1, N2) and transition phase during trot-walk (n = 325) and walk-trot (n = 299) transitions. Data were collected at two locations (S, N). For each teacher two lessons with each of five students were recorded (10 lessons per teacher), with partial crossover of horses and riders within location. Results from mixed models with teacher, transition phase and their interaction as fixed effects and horse, rider and lesson serial number as random effects.

		Riding teacher							
Transition		S1 (Anna)		S2 (Bella)		N1 (Cecilia)		N2 (Diana)	
	Phase	Est	SE	Est	SE	Est	SE	Est	SE
Trot-walk	Preparation	7.31	0.17	7.58	0.17	7.55	0.18	7.61	0.19
	Transition	1.75	0.17	1.58	0.17	2.18	0.18	1.90	0.19
	Post-transition	3.71	0.17	3.85	0.17	4.41	0.18	7.13	0.19
Walk-trot	Preparation	3.39	0.17	3.74	0.16	4.08	0.17	7.11	0.18
	Transition	1.96	0.17	1.95	0.16	2.26	0.17	2.05	0.18
	Post-transition	7.88	0.17	7.92	0.16	7.90	0.17	7.83	0.18

#### 3.4.2 Differences in horse and rider biomechanics between riding teachers

There was a significant interaction between teacher and transition phase for twelve of thirteen horse and rider movement variables. For those variables, least square means per teacher and transition phase can be found in Table 4; standard errors and p-values for post hoc pairwise comparisons between different teachers can be found in S3 Table. Hind limb protraction consistency, the remaining variable, showed a significant main effect of teacher (p < 0.05) but only during up-transitions (S2 Table).

**Table 4:** **Horse and rider motion and rein tension variables for a) down-transitions, and b) up-transitions, between walk and trot.** Least square means from mixed models by riding teacher (S1, S2, N1, N2) and transition phase (preparation, transition, post-transition) during 325 trot-walk and 299 walk-trot transitions, respectively. Rein tension is the left-right rein average unless stated otherwise. Data were collected at two locations (S, N). For each teacher two lessons with each of five students were recorded (10 lessons per teacher), with partial crossover of horses and riders within location. The models were based on stride data (trot-walk transitions: n = 4518 strides; walk-trot transitions: n = 4295 strides; protraction-retraction data were missing for one lesson), and included teacher, transition phase and their interaction as fixed effects and horse, rider and transition serial number nested in lesson serial number as random effects. The interaction between teacher and transition phase was significant (p < 0.05) for all variables listed. Bolded numbers indicate significant differences (post-hoc comparisons) between teachers at the same location (pair S, pair N).

		Pair S		Pair N	
a) Trot-walk transitions	Phase	S1Anna	S2Bella	N1Cecilia	N2Diana
Forelimb protraction-	Prep	76.2	76.3	76.4	77.3
retraction ROM	Tran	67.4	68.6	67.9	69.0
(°)	Post	**70.6**	**73.4**	69.9	70.0
Hind limb protraction-	Prep	51.0	50.8	**49.0**	**49.6**
retraction ROM	Tran	**55.1**	**57.1**	**52.5**	**53.8**
(°)	Post	**56.6**	**57.8**	**55.7**	**54.7**
Hind limb maximum	Prep	**30.3**	**31.1**	**29.0**	**28.5**
protraction	Tran	**29.3**	**31.1**	28.5	28.4
(°)	Post	**29.6**	**30.8**	28.4	29.6
Hind limb maximum	Prep	**−20.6**	**−19.7**	−20.0	−20.0
retraction	Tran	−25.7	−25.9	**−24.0**	**−25.3**
(°)	Post	−27.1	−27.0	**−27.2**	**−26.7**
Absolute left-right difference	Prep	**1.8**	**1.6**	1.7	1.6
hind limb maximum	Tran	3.3	3.3	2.9	3.2
Retraction∗ (°)	Post	1.7	1.7	**1.5**	**1.3**
Minimum rein tension∗	Prep	6.9	7.3	7.8	7.8
(N)	Tran	**5.2**	**4.0**	5.0	5.2
	Post	**3.7**	**2.5**	**3.4**	**4.3**
Median rein tension∗	Prep	**20.8**	**24.7**	23.9	22.4
(N)	Tran	16.5	14.7	18.3	17.5
	Post	**13.5**	**9.9**	14.9	15.4
Maximum rein tension∗	Prep	**37.3**	**57.5**	**55.8**	**49.5**
(N)	Tran	**30.5**	**36.2**	**43.2**	**36.0**
	Post	27.4	26.0	37.0	34.5
Absolute left-right difference	Prep	**3.3**	**5.3**	5.9	5.3
median rein tension∗	Tran	2.8	3.4	5.4	5.1
(N)	Post	2.7	2.6	**5.8**	**4.7**
Phase (% of stride) between	Prep	**1.8**	**0.6**	0.1	0.3
sacrum and withers	Tran	**3.3**	**5.4**	4.1	3.1
vertical excursion∗	Post	**1.7**	**17.1**	13.9	13.9
Rider sacrum roll ROM	Prep	**6.23**	**5.69**	**4.80**	**5.08**
(°)	Tran	**7.87**	**8.34**	**6.58**	**7.27**
	Post	**6.75**	**7.34**	6.79	6.97
Rider sacrum pitch ROM∗	Prep	19.0	18.7	15.7	15.0
(°)	Tran	**12.4**	**14.4**	**15.8**	**14.3**
	Post	**8.4**	**9.7**	**10.1**	**9.5**

		Pair S		Pair N	
**b) Walk-trot transitions**	Phase	S1Anna	S2Bella	N1Cecilia	N2Diana
Forelimb protraction-	Prep	**70.8**	**73.3**	**69.2**	**69.9**
retraction ROM	Tran	**73.2**	**74.1**	**71.5**	**72.6**
(°)	Post	77.5	75.5	77.1	77.7
Hind limb protraction-	Prep	**56.5**	**58.0**	55.3	54.2
retraction ROM	Tran	**53.2**	**54.2**	**51.9**	**53.5**
(°)	Post	**53.4**	**52.4**	**51.5**	**52.1**
Hind limb maximum	Prep	29.2	31.1	**28.2**	**27.8**
protraction	Tran	28.2	28.9	27.1	27.3
(°)	Post	31.7	31.9	**30.5**	**31.0**
Hind limb maximum	Prep	−27.2	−26.9	**−27.1**	**−26.4**
retraction	Tran	−24.9	−25.4	**−24.8**	**−26.2**
(°)	Post	**−21.7**	**−20.6**	−21.0	−21.2
Absolute left-right difference	Prep	1.7	1.6	**1.7**	**1.2**
hind limb maximum	Tran	3.2	3.8	**2.9**	**2.3**
retraction∗ (°)	Post	1.6	1.6	1.6	1.6
Minimum rein tension∗	Prep	**3.5**	**2.4**	3.5	3.9
(N)	Tran	3.2	3.1	3.4	4.4
	Post	3.7	3.4	**3.1**	**4.4**
Median rein tension∗	Prep	**13.1**	**9.8**	15.0	14.3
(N)	Tran	13.0	12.9	14.2	14.5
	Post	15.0	16.4	16.5	16.4
Maximum rein tension∗	Prep	26.7	26.5	**37.0**	**32.9**
(N)	Tran	29.6	31.6	42.0	37.9
	Post	**30.0**	**40.3**	47.3	45.4
Absolute left-right difference	Prep	2.5	2.9	**5.4**	**5.3**
median rein tension∗	Tran	**3.0**	**4.6**	5.4	6.1
(N)	Post	**3.7**	**5.3**	**6.1**	**5.1**
Phase (% of stride) between	Prep	**7.7**	**16.4**	12.9	13.2
sacrum and withers	Tran	**−0.19**	**−3.22**	−1.31	−0.88
vertical excursion∗	Post	0.86	0.71	0.00	0.00
Rider sacrum roll ROM	Prep	**6.69**	**7.32**	6.62	6.53
(°)	Tran	5.53	5.22	**4.24**	**5.07**
	Post	**6.30**	**5.78**	**4.71**	**5.18**
Rider sacrum pitch ROM∗	Prep	**8.8**	**9.6**	**10.5**	**8.9**
(°)	Tran	13.8	13.5	11.2	10.6
	Post	19.7	19.4	15.7	15.8

∗ back-transformed estimates.

In the pairwise comparisons between the teachers, Anna (S1) and Bella (S2), the two teachers at location S (pair S), differed most frequently, followed by the two teachers at location N (pair N), Cecilia (N1) and Diana (N2). Significant differences between teachers at different locations were least common and the p-values for those comparisons were generally higher (often 0.01–0.05) than for significant comparisons between teachers at the same location. Among the significant between-location comparisons, Anna and Cecilia differed most frequently during down-transitions, whereas for up-transitions Anna differed near equally often from Cecilia and from Diana. Significant differences between teachers are further detailed below (0.05 > p > 0.0001 where not stated, p-values can be found in S3 Table), and significant differences for pair S (S1 vs S2) and pair N (N1 vs N2), respectively, are indicated in bold in Table 4.

During Bella's lessons, the horses moved with greater forelimb protraction-retraction ROM (range of motion) in walk (p < 0.0001) and during up-transitions when comparing with Anna's lessons with the same riders and horses. Further, hind limb protraction-retraction ROM was greater both in walk and during transitions (p = 0.005-<0.0001), and hind limb maximum protraction was greater throughout down-transitions (p = 0.0003-<0.0001). With Bella, horses also displayed better hind limb retraction consistency in trot during preparation for a down-transition and median rein tension was lower in walk (p < 0.0001). The expected relative phase between the vertical movement of the withers and of the croup is 0 % in trot and 25 % in walk, but model estimates for walk were below 25 % for all teachers, suggesting a residual and/or anticipatory “trot” effect on walk mechanics. The relative phase was closer to the expected value in both trot and walk (walk p < 0.0001) during lessons with Bella (trot 0.6–0.7 %, walk 16–17 %) vs Anna (trot 1–2 %, walk 9 %). In walk, the phase shift was closer to the expected for Bella compared to all other teachers (p = 0.04-<0.0001). Compared to Bella, with Anna horses showed greater hind limb maximum retraction (which follows the propulsive phase of the stride, smaller angle means greater retraction) in trot (p < 0.0001). Further, with Anna maximum rein tension was lower in trot than with Bella. This was especially true during preparation for down-transitions (Anna 37.3 N, Bella 57.5 N, p < 0.0001), while the difference was smaller following the down-transitions (Anna 30.0 N, Bella 40.3 N, p < 0.0001). Maximum rein tension for Anna was also lower compared to Cecilia during down-transitions (Anna 30.5 N, Cecilia 43.2 N) and in trot before the down-transitions (Anna 37.3 N, Cecilia 55.8 N) and following the up-transitions (Anna 30.0 N, Cecilia 47.3 N). With Anna, riders had more symmetric rein tension than with Bella in trot (p < 0.0001) and during up-transitions. Rider pelvic roll ROM generally tended to be slightly higher during down-transitions, and slightly lower during up-transitions, compared to both walk and trot. Comparing Anna and Bella, rider pelvic roll ROM was relatively larger in trot but smaller in walk with Anna, whereas with Bella the riders had larger pelvic pitch ROM during down-transitions and in walk.

Comparing pair N (Cecilia and Diana), hind limb ROM was slightly larger before and larger during down-transitions in lessons with Diana than with Cecilia. Both fore- and hind limb ROM were larger with Diana during (hind ROM p < 0.0001) and after up-transitions than with Cecilia. Additionally, the horses showed greater hind limb maximum retraction during both down- and up-transitions (up p < 0.0001), and slightly better hind limb protraction consistency overall (smaller stride-to-stride difference; back-transformed estimate Diana 2.0° vs Cecilia 2.3°, S2 Table) with Diana. Finally, with Diana horses showed better hind limb retraction consistency in walk (p < 0.0001) and during up-transitions compared to Cecilia (Table 4), as well as compared to the other teachers (S3 Table). Further, maximum rein tension was lower with Diana than with Cecilia before (Cecilia 55.8 N, Diana 49.5 N) and during down-transitions (Cecilia 43.2 N, Diana 36.0 N). During lessons with Cecilia, on the other hand, the horses showed larger hind limb ROM and greater hind limb maximum retraction in walk. Rider pelvic roll ROM was slightly larger in trot and during transitions with Diana than with Cecilia, whereas rider pelvic pitch ROM was larger in walk (p < 0.0001) and during down-transitions with Cecilia.

## 4 Discussion

The current study explores what insights can be gained about teaching the embodied knowledge of riding from combining qualitative methods (interviews and conversation analysis) and quantitative measurements (horse and rider motion and rein tension). Reviewing the results from this combined perspective, differences in horse and rider movements and rein tension during lessons with different riding teachers appear to reflect differences in the lesson structure and instruction focus. The results confirm that an experienced riding teacher can have a consistent influence across a group of riders, at least if the riders have a similar skill level.

### 4.1 Instruction focus

Teachers Bella (S2) and Diana (N2) had a relatively greater focus on the walk, in comparison with Anna (S1) and Cecilia (N1), who focused more on that the horse should be quick, energetic and attentive, ready to transition back to trot after only a few walk strides. Bella asked the riders to feel and count each walk stride between down- and up-transitions. At the same time, her students achieved walk with a phase shift (relative timing within the stride cycle) of 16–17 % between the up-down movement of the withers and of the croup, closer to the ideal 25 % [44] than for any other riding teacher. Diana asked for longer walk sequences in-between transitions and commented more on the walk rhythm than the others. At the same time, the average stride-to-stride difference in hind limb retraction in walk was 1.2–1.3° during Diana's lessons, compared to 1.5–1.7° for the other teachers, which suggests that the walk was more symmetric and regular. Limb coordination asymmetries in walk are previously known to occur in dressage horses, possibly related to limb preference, while straightness and symmetry are considered primary goals in dressage training [45,46]. These findings suggest that what aspects the teacher chooses to focus on affect horse and rider performance, as the teachers that focused more on the walk, the walk rhythm was better. This concept could be further explored in intervention studies.

### 4.2 Lesson design

Bella and Cecilia asked their riders to start make transitions between walk and trot on straight lines from the beginning of their lessons. In contrast, Anna and Diana spent a third or more of their lessons preparing the students for the given task. Anna started with transitions on the circle and gradually approached the long side, and Diana started with walk-halt transitions. When their students subsequently performed trot-walk transitions on the long side, maximum rein tension just before and during down-transitions was lower compared to the other two teachers, suggesting that their respective strategy was effective in preparing the riders and their horses. During preparation for down-transitions the difference in maximum rein tension between Bella and Anna was 20 N per rein on average, which corresponds to approximately 4 kg of extra load in the horse's mouth. A similar but smaller difference was observed comparing Cecilia to Diana. This may suggest that the riders struggled more in their communication with the horse during the transitions when these were less prepared and felt compelled to use harsher rein tension signals (consciously or unconsciously) to accomplish their goals [29]. All teachers similarly encouraged the riders to use primarily their seat and legs to control the tempo. Thus, it seems that thoughtful chaining of exercises during the lesson can be equally or more important for achieving lighter rein tension, as opposed to instructions given during the exercise, at least within the context of a single lesson.

Bella and Cecilia still achieved good results with their students, though. During the lessons with Bella, the horses showed greater protraction-retraction ROM in walk and during up-transitions, suggesting a longer stride length [47], a relative timing between the withers and the croup closer to 25 %, and greater hind limb protraction consistency in trot preparing for a down-transition, compared with Anna. Cecilia's students similarly achieved larger hind limb ROM and greater maximum hind limb protraction in walk, suggesting longer stride length and better forward reach of the hind limbs [47], compared to during lessons with Diana. This illustrates that teaching riding is indeed a complex task, where achieving some goals can be in opposition to achieving others.

### 4.3 Implications for riding teacher education

Majlesi and Broth [48] explored how different learnables emerge in the interaction between teacher and learner. They defined a learnable as a shared pedagogic focus that both student and teacher finds relevant. This is an interesting way to explain how riding is taught and learned. It is not a linear process, as each riding lesson presents different situations. A riding teacher needs to consider not only how to improve the performance of the horse-rider pair and achieve progress within their education, but also how to improve the confidence and welfare of both horse and rider. Even though the riding teachers were all were asked to hold 20-min lessons with a focus on transitions on one long side, they planned and carried out their lessons in four different ways. This reveals the gap of knowledge in the optimal way to give a riding lesson (e.g. Ref. [3]) and shows that riding teachers do not necessarily share a common base for how to teach riding. Rather, a substantial part of a riding teacher's professional knowledge is based on individual experience.

For several different sports, including riding, studies have indicated that augmented feedback, information provided to the learner from an external source, can improve performance and learning of sports-specific skills [49,50]. In the context of teaching, instructions and evaluations based on observation of students' learning outcomes (instructed actions) are considered cornerstones in order for a teacher to guide a learner through the process of turning experiences into knowledge [51]. Observing the students' learning outcomes in relation to the given instructions is equally important for teachers to develop in their role. Quantitative measurements could be supportive to this, as such data can highlight aspects that the teacher might be unaware of. While the observed differences in horse and rider movement patterns and rein tension magnitude between the riding teachers appear related to intentional choices, it is less clear to what extent these specific, measurable effects were consciously intended. Bella and Diana indeed actively focused on the quality of the walk. However, based on what they emphasised during the interviews before and after the lessons, Anna and Diana did not choose their strategy for preparing the transitions specifically to achieve lower rein tension. Knowledge that this was the outcome could make them (even) more inclined to continue to use this strategy in the future and could encourage other riding teachers to do the same. In general, increased reflection and feedback on the effects and consequences of certain choices could be supportive to riding teacher's professional development. In the future, quantitative measurements – together with an understanding of the relation between measured data, instructions and riders' equestrian feel – could become a tool in both riding teacher education and during ordinary riding lessons.

### 4.4 Learning during riding lessons

A recurrent theme during interviews with riding teachers, both in the present and in previous studies, is the question of whether to focus more on understanding or on performing. One could say that riding is a practical skill and the only way to learn it is to ride, which the riding teachers interviewed in Lundesjö Kvart (2020a, p173) underline. On the other hand, the importance of discussion and reflection was mentioned by the riding teachers both in the current and in previous studies [22,23]. This can be understood to reflect a wish that the riders should understand the task and not merely follow orders. Similarly, Evans [52] shows how coaches work with their basketball players (a game which requires practical skills, as is true for riding) to make them understand the plan of the game by discussing different ways to make an offensive attempt towards the other team. Since the study period was brief and without a follow-up, it is not possible to determine what the riders took away from the lessons. However, horse and rider movements and rein tension during the lessons do reflect the riders' actions and can thereby provide some clues to how the riding teachers' intentions and instructions were understood by the riders. In a further analysis of the collected material, we aim to investigate differences between consecutive transitions and associations with the teachers’ evaluations, to explore both perceived and objective progression during the lessons.

### 4.5 Strengths and limitations

The interdisciplinary, mixed methods approach taken in this study produce and provided valuable insights and presents a novel approach to studying teaching of riding. However, this is not to say that the analyses were optimal or exhaustive. Similar to most interdisciplinary studies on sports performance [26], the current study focused on the group level differences and general patterns, and the results of the quantitative and qualitative analyses were integrated at this level. In some sense, the conversational analysis and interview results were adapted to the common paradigm for analysing biomechanical data. Future studies may explore alternative integration perspectives, for example attempting to integrate the results on a case-by-case level.

The number of subjects included in the study was small, but average compared to other equine biomechanics studies [53]. The riders at each location all had similar training, and the school horses also represent a selected group compared to horses in general. This limits the general applicability of the results. Repeating the study in a different setting, as well as conducting a larger experimental study designed to test the core conclusions from this study, would be valuable for evaluating how well the study findings hold in a wider context.

Comparisons between each pair of teachers at the same location were more frequently statistically significant, despite that differences for the biomechanical variables were not numerically larger in general than differences between teachers at different locations. This could possibly reflect that individual variation among horses and riders was better accounted for within location, where there was a partial crossover, compared to between locations.

The different methodological approaches, including the avoidance of bias across methodologies by applying them separately to the collected research material, have been crucial for a relevant interpretation of the data and results. Each author was responsible for their part of the analysis initially, and in a second phase, the results were combined and compared. With this interdisciplinary approach, both qualitative and quantitative aspects have been made visible.

## 5 Conclusion

The current study found that riding teachers have an influence on the movement pattern of the horses and riders they teach, as well as on the magnitude of rein tension. While likely unsurprising to most equestrians, the current study is, to the authors' knowledge, the first to quantify this. In particular, that more preparation of the students led to lighter rein aids during transitions highlight the importance of lesson planning: it's not only about what you ask the students to do, or how, it's also about the order and relative difficulty of consecutive exercises. This presents both opportunity and responsibility for riding teachers and opens for further research on how to safeguard and improve welfare for riding horses while also achieving good performance and effective learning.

## CRediT authorship contribution statement

**Anna Byström:** Conceptualisation, Funding acquisition, Data curation, Formal analysis, Writing – original draft, Writing – review & editing. **Agneta Egenvall:** Funding acquisition, Investigation, Formal analysis, Writing – original draft, Writing – review & editing. **Marie Eisersiö:** Funding acquisition, Investigation, Writing – review & editing. **Maria Terese Engell:** Funding acquisition, Investigation, Writing – review & editing. **Sigrid Lykken:** Funding acquisition, Writing – review & editing. **Susanne Lundesjö Kvart:** Conceptualisation, Funding acquisition, Investigation, Data curation, Formal analysis, Writing – original draft, Writing – review & editing.

## Data availability statement

Horse and rider motion and rein tension data used for the statistical analysis are available in S3 File. Transcripts from the interviews will not be shared in the interest of the participants’ privacy and anonymity. Transcripts from the analysed parts of the riding lessons can be retrieved from the authors upon request (in original language, i.e. Swedish or Norwegian).

## Funding

The study received funding from the 10.13039/100031135Swedish-Norwegian Foundation for Equine Research, grant numberH-20-47-567 (granted to all authors, main applicant Anna Byström). The funders had no role in study design, data collection and analysis, decision to publish, or preparation of the manuscript.

## Declaration of competing interest

The authors declare that they have no known competing financial interests or personal relationships that could have appeared to influence the work reported in this paper.
