# Supplementary material for: The impact of teaching approach on horse and rider biomechanics during riding lessons
Source: Heliyon. 2025 Jan 14;11(2):e41947. doi: 10.1016/j.heliyon.2025.e41947 (PMC11791127; doi:10.1016/j.heliyon.2025.e41947)
Supplement: Multimedia component 1 [file mmc1.pdf]

Interview templates – Equestrian feel in practice

The purpose of the project is to study how riding teachers instruct the rider in moments where the rider must give the horse aid so that he can perform a specific movement/exercise.

Riding teacher before lesson:
(30 min, more general questions)
How do you work with instructions during riding lessons? (*when do you give, intensity, content, check if it was understood, optimal time to give in order to reach...*)

How do you think about the timing of instructions to the rider relative to the timing of the rider giving aids to the horse?
What is equestrian feel to you?
How do you think about the riding teacher's role in the rider's development of equestrian feel?
How do you see the interaction between teacher-rider-horse?
How do you feel that your horses are able to respond to/understand the riders' aids and perform desired movements?

(if there is time, otherwise at the end of the day...)
Is learning theory and positive or negative reinforcement something you actively think about in your teaching?
Can you describe some common "errors/problems" in the rider's aids and the horses' following movements/actions, and how you usually correct them?
Are there particular moments where you feel that timing is a common problem (*rider's aids come too late or discordant to the horse*)?

Riding teacher after lesson:
With film clips from each rider's lessons. Select some interesting clips/students based on the student interviews. (Total 60 min, approx. 10 min/student clip)

What do you perceive of the horse and rider here? What is happening?
Describe what you see/how you thought…
How did you think about the instructions you gave?
How did the rider manage to carry out your instructions, where the rider's *actions/actions/signals/execution* timely?
What effect did the instruction have on the horse?
How was the timing of instructions relative to the execution of the movement/exercise?

(If there is time for each student, otherwise at the end)
Which (type of) instruction was/is helpful in which situation?
Describe how you perceive the rider's ability to give well-timed aids to the horse.
Did you see any development/progression during the lesson?
How do you perceive the rider and horse communication?

Rider after the lessons:
(20 min/student, first short general reflection then watch film clip)

How did it go today? (*horse 1/horse 2*)
(start watching film clips) What's going on here…
How did you experience this situation from horseback/how do you perceive that the horse reacted?
What did you perceive that the riding teacher instructed (*said*)?
How did you understand that instruction?

  (ask when it fits)
  How much do you have time to think about the instructions you receive during an ongoing riding exercise?
  How would you like to receive instructions while riding?
  What kind of instruction is helpful in what situation?

What did you do after the instruction?
Did you manage to help the horse in time?

  (ask when it fits)
  How did you feel that you were given instructions on when and how to give your aids?
  When is the optimal time for you to receive instructions relative to your interaction with the horse?
  How do you think about your communication with horse 1/horse 2?

Did the horse do what you wanted it to do?
In what ways did you get help…

  to perceive signals from the horse?
  to observe the horse's movements?
  to pay attention to how different situations felt? (*ask when it fits*)

(If there is time)
What is equestrian feel to you?
How do you think about the riding instructor's role in the rider's development of equestrian feel?
What is your view on the interaction between teacher-rider-horse?
Is learning theory and positive or negative reinforcement something you actively think about your riding?

Intervjumallar – Ryttarkänsla i praktiken

Syfte med projektet är att studera hur ridlärare instruerar ryttaren i moment där ryttaren ska ge hästen hjälper så denne kan utföra en specifik rörelse/övning.

Ridlärare före lektion:
(30 min, mer övergripande frågor)
Hur arbetar du med instruktioner under ridlektioner? (*när ger du, intensitet, innehåll, stämma av att du nått fram, optimal tid ge för att nå fram…)*

Hur tänker du kring tajming av instruktioner till ryttaren relativt tajming av ryttarens hjälpgivning till hästen?
Vad är ryttarkänsla för dig?
Hur tänker du kring ridlärarens roll i ryttares utveckling av ryttarkänsla?
Hur ser du på samspelet mellan lärare-ryttare-häst?
Hur upplever du att era hästar klarar av att svara på/förstå ryttarnas hjälper och utföra önskade rörelser?

(om det hinns med, annars i slutet på dagen…) Är inlärningsteori och detta med positiv respektive negativ förstärkning något du aktivt tänker på i din undervisning?
Kan du beskriva några vanliga ”fel/problem” i ryttares hjälpgivning och hästarnas påföljande rörelser/agerande, samt hur du brukar korrigera dem?
Finns det speciella moment där du upplever att timing är ett vanligt problem (*ryttarens hjälper kommer för sent eller i otakt med hästen*)?

Ridlärare efter lektion:
Till filmklipp från resp ryttares lektioner. Välja ut några intressanta klipp/elev utifrån elevintervjuerna. (Totalt 60 min, ca 10 min/elevs klipp)

Vad är det du uppfattar på ekipaget här? Vad händer?
Berätta vad du ser/hur du tänkte…
Hur tänkte du kring instruktionerna du gav?
Hur lyckades ryttaren utföra dina instruktioner, kom deras *agerande/ handlingar/ signaler/ utföranden* i rätt tid?
Vad fick instruktionen för effekt på hästen?
Hur var tajmingen av instruktioner relativt rörelsernas utförande?

(*Om det hinns med för respektive elev, annars på slutet*)
Vilken (typ av) instruktion var/är hjälpsam i vilken situation?
Beskriv hur du uppfattar ryttarens förmåga att ge väl tajmade hjälper till hästen?
Såg du någon utveckling/progression under lektionen?
Hur uppfattar du ryttarens och hästens kommunikation?

Ryttare efter lektion:
(20 min/elev, först kort allmän reflektion, sidan titta på filmklipp)

Hur gick det idag? (*häst 1/häst2*)
(*börja titta på filmklipp*) Vad händer här…
Hur upplevde du den här situationen från hästryggen/hur reagerade hästen tycker du?
Vad uppfattade du att ridläraren instruerade (*sa*)?
Hur förstod du den instruktionen?
(*fråga när det passar in*)
Hur mycket hinner du tänka kring instruktioner du får under pågående ridövning?
Hur vill du ha instruktioner under pågående ridning?
Vilken sorts instruktion är hjälpsam i vilken situation?
Vad gjorde du efter instruktionen?
Hann du utföra hjälpen till hästen i tid upplevde du?
(*fråga när det passar in*)
Hur upplevde du att du fick instruktioner om när och hur du skulle ge dina hjälper?
När är optimal tidpunkt för dig att få instruktioner i förhållande till din interaktion med hästen?
Hur tänker du kring din kommunikation med häst 1/häst2?
Gjorde hästen det du ville den skulle göra?
På vilka sätt fick du hjälp…
att uppfatta signaler från hästen?
att observera hästens rörelser?
att uppmärksamma hur olika situationer kändes? (*fråga när det passar in*)

(*Om det hinns med*)
Vad är ryttarkänsla för dig?
Hur tänker du kring ridlärarens roll för ryttares utveckling av ryttarkänsla?
Hur ser du på samspelet mellan lärare-ryttare-häst?
Är inlärningsteori och detta med positiv resp negativ förstärkning något du aktivt tänker på i din ridning?
