# Supplementary material for: The impact of teaching approach on horse and rider biomechanics during riding lessons
Source: Heliyon. 2025 Jan 14;11(2):e41947. doi: 10.1016/j.heliyon.2025.e41947 (PMC11791127; doi:10.1016/j.heliyon.2025.e41947)
Supplement: Multimedia component 4 [file mmc4.docx]

**S1 Table. Horse age, height and equipment.**

| No. | Country | Age | Gender | Withers  height (cm) | Bridle | Nose band |
| --- | --- | --- | --- | --- | --- | --- |
| 1 | SE | 12 | Mare | 163 | Single-jointed snaffle, loose rings | Cavesson noseband with flash |
| 2 | SE | 9 | Gelding | 167 | Single-jointed snaffle, loose rings | Cavesson noseband with flash |
| 3 | SE | 16 | Gelding | 168 | Straight mouth-piece, fixed rings | Drop noseband |
| 4 | SE | 14 | Mare | 165 | Single-jointed snaffle, loose rings | Cavesson noseband with flash |
| 5 | SE | 7 | Gelding | 165 | Double-jointed snaffle, loose rings | Cavesson noseband with flash |
| 11 | SE | 19 | Gelding | 170 | Single-jointed rubber snaffle (day 1) Double-jointed snaffle, loose rings(day 2) | Cavesson noseband with flash |
| 6 | NO | 11 | Gelding | 170 | Double-jointed snaffle, loose rings | Cavesson noseband with flash |
| 7 | NO | 8 | Mare | 162 | Double-jointed snaffle, loose rings | Cavesson noseband with flash |
| 8 | NO | 6 | Gelding | 173 | Double-jointed snaffle, loose rings | Micklem bridle |
| 9 | NO | 11 | Gelding | 165 | Double-jointed snaffle, loose rings | Cavesson noseband with flash |
| 10 | NO | 14 | Mare | 168 | Double-jointed snaffle, loose rings | Cavesson noseband with flash |
