# Supplementary material for: The impact of teaching approach on horse and rider biomechanics during riding lessons
Source: Heliyon. 2025 Jan 14;11(2):e41947. doi: 10.1016/j.heliyon.2025.e41947 (PMC11791127; doi:10.1016/j.heliyon.2025.e41947)
Supplement: Multimedia component 5 [file mmc5.docx]

**S2 Table. Hind limb protraction consistency during up-transitions.** Least square means (Est) from mixed models with standard error (SE) and back-transformed (BT) values, by riding teacher (S1, S2, N1, N2) during 299 walk-trot transitions (n=4295 strides), respectively, along with p-values for pairwise comparisons between teachers. Data were collected at two locations (S, N). For each teacher two lessons with each of five students were recorded (10 lessons per teacher), with partial crossover of horses and riders within location. The models included teacher as fixed effects and horse, rider and transition serial number nested in lesson serial number as random effects (transition phase and phase × teacher interaction were tested but were not significant).

| **Teacher** | **Est** | **SE** | **BT** |
| --- | --- | --- | --- |
| S1 Anna | -0.74 | 0.008 | 2.33 |
| S2 Bella | -0.75 | 0.008 | 2.20 |
| N1 Cecilia | -0.74 | 0.009 | 2.25 |
| N2 Diana | -0.76 | 0.009 | 2.01 |
|  |  |  |  |
| **Differences between teachers** | | | |
|  |  | **p-value** |  |
| S1 | S2 |  |  |
| S1 | N1 |  |  |
| S1 | N2 |  |  |
| S2 | N1 |  |  |
| S2 | N2 |  |  |
| N1 | N2 | 0.006 |  |
