# Supplementary material for: The impact of teaching approach on horse and rider biomechanics during riding lessons
Source: Heliyon. 2025 Jan 14;11(2):e41947. doi: 10.1016/j.heliyon.2025.e41947 (PMC11791127; doi:10.1016/j.heliyon.2025.e41947)
Supplement: Multimedia component 7 [file mmc7.pdf]

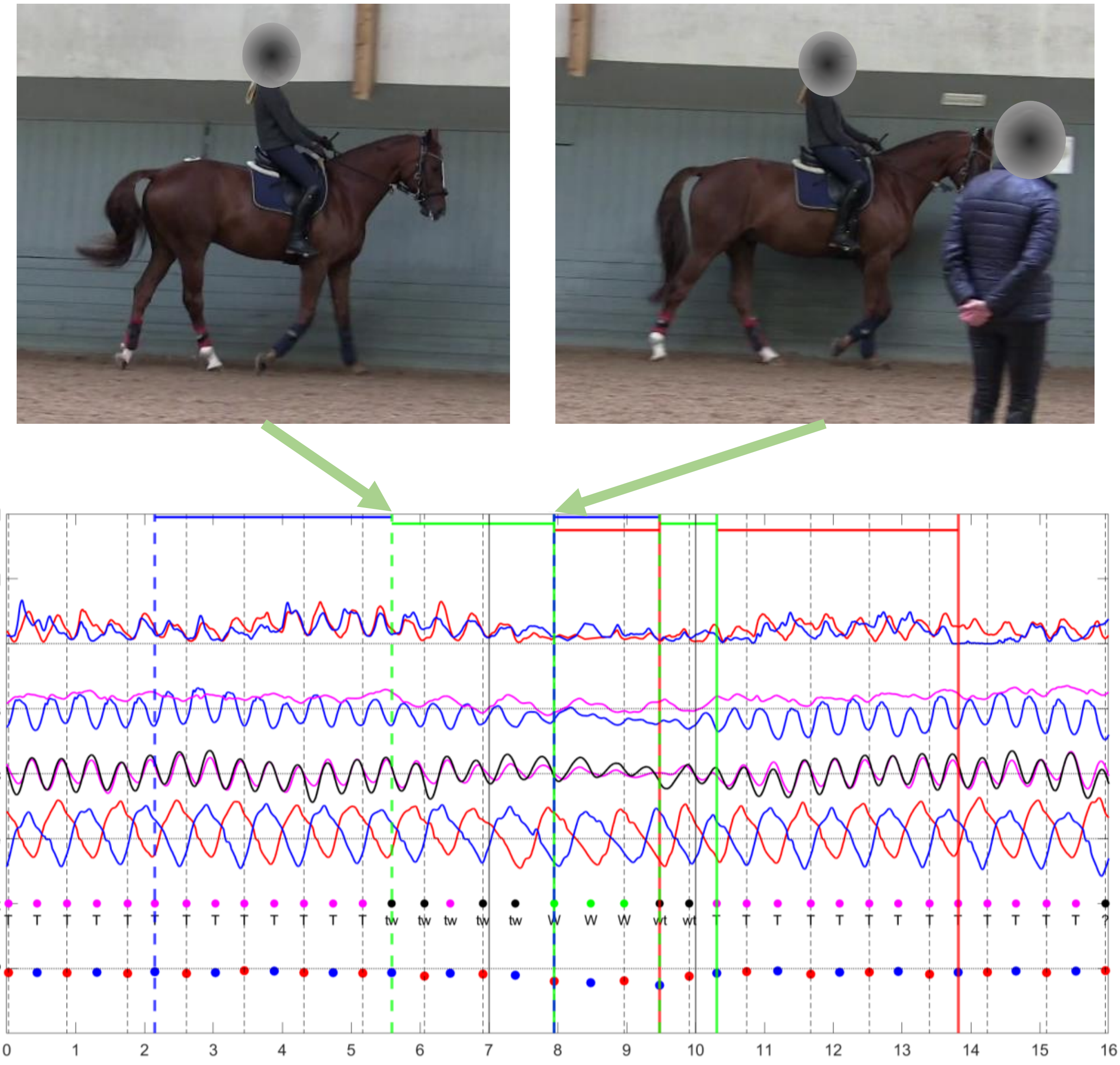

S1 Fig. Example of rein tension and horse and rider movement data with images from the synchronised video recording. One down- (trot-walk) and one up-transition (walk-trot) in right direction. Top row: rein tension (blue right rein, red left rein). 2nd row: rider pelvic roll (side-to-side rotation, blue, positive to the right) and pitch (forward-backward rotation, magenta, positive forwards). 3rd row: vertical translation of the horse's withers (magenta) and croup (black). 4th row: protraction-retraction of the hind limbs (blue right, red left, positive for forward reach). 5th row: gait classification based on limb timing (magenta trot, black undefined/transitional stride, green walk) and letters to indicate transitions (tw trot-walk, wt walk-trot). Bottom row: hind limb diagonal advanced placement relative the diagonal forelimb (time delay at hoof on, blue right hind, red left hind). Vertical interrupted lines indicate touchdown of the left hind limb. Vertical blue (preparation) green (transition) and red (post-transition) show the data selected for each transition phase. Vertical grey solid lines indicate when transitions were visually perceived to occur. The y-scale is normalized for display, except for rein tension; curves on the same row are on the same scale and can be compared. The x-scale is in seconds (set to zero at the start of the displayed sequence for ease of reading).
